# Fast protein structure comparison through effective representation learning with contrastive graph neural networks

**DOI:** 10.1371/journal.pcbi.1009986

**Published:** 2022-03-24

**Authors:** Chunqiu Xia, Shi-Hao Feng, Ying Xia, Xiaoyong Pan, Hong-Bin Shen

**Affiliations:** Institute of Image Processing and Pattern Recognition, Shanghai Jiao Tong University, and Key Laboratory of System Control and Information Processing, Ministry of Education of China, Shanghai, China; San Raffaele Hospital: IRCCS Ospedale San Raffaele, ITALY

## Abstract

Protein structure alignment algorithms are often time-consuming, resulting in challenges for large-scale protein structure similarity-based retrieval. There is an urgent need for more efficient structure comparison approaches as the number of protein structures increases rapidly. In this paper, we propose an effective graph-based protein structure representation learning method, GraSR, for fast and accurate structure comparison. In GraSR, a graph is constructed based on the intra-residue distance derived from the tertiary structure. Then, deep graph neural networks (GNNs) with a short-cut connection learn graph representations of the tertiary structures under a contrastive learning framework. To further improve GraSR, a novel dynamic training data partition strategy and length-scaling cosine distance are introduced. We objectively evaluate our method GraSR on SCOPe v2.07 and a new released independent test set from PDB database with a designed comprehensive performance metric. Compared with other state-of-the-art methods, GraSR achieves about 7%-10% improvement on two benchmark datasets. GraSR is also much faster than alignment-based methods. We dig into the model and observe that the superiority of GraSR is mainly brought by the learned discriminative residue-level and global descriptors. The web-server and source code of GraSR are freely available at www.csbio.sjtu.edu.cn/bioinf/GraSR/ for academic use.

## Author summary

The size and shape of protein structures vary considerably. Accurate protein structure comparison usually relies on structure alignment algorithms. However, superimposing two protein structures is relatively time-consuming, which makes it inappropriate for large-scale protein structure retrieval. Alignment-free algorithms are proposed for efficient protein structure comparison over the last few decades. These algorithms first transform the coordinates of atoms in two proteins to fixed-length vectors. Then, the comparison can be done by measuring the distance or similarity between two vectors, which is much faster than alignment. In this study, we propose a novel protein structure representation method for efficient structure comparison. Compared with other state-of-the-art alignment-free methods, our method achieves better performance on both ranking and multi-class classification tasks due to the powerful representation ability of deep graph neural networks. We dig into the model and observe that the superiority of our method is mainly brought by the learned discriminative residue-level and global descriptors.

This is a *PLOS Computational Biology* Software paper.

## Introduction

Protein structure comparison aims to measure the structural similarity between two different proteins. It is a core infrastructure for structural biology and provides support for protein structure prediction [1], protein-protein docking [2], structure-based protein function prediction [3], etc. Considering the number of experimentally solved protein structures is increasing rapidly in the Protein Data Bank (PDB) and the accuracy of protein structure prediction has improved dramatically in recent years, e.g. AlphaFold2 approach [4], it is highly desired to develop fast and accurate protein tertiary structure comparison methods which could benefit structural homology discovery and other downstream structure-based analysis [5].

Protein structure comparison methods can be generally divided into two types: alignment-based methods [6–13] and alignment-free methods [14–16]. The former finds the optimal structural superposition of two protein structures. Then, scoring functions, such as RMSD (root-mean-squared deviation) [17], are used to measure the Euclidean distance between each pair of corresponding residues in the two proteins. The latter usually first transforms the Cartesian coordinates of all backbone atoms of a protein structure to a vector. Then, the structural comparison is performed by calculating the distance or correlation coefficient between the two corresponding vectors.

For alignment-based methods, the most challenging task is how to superimpose the atomic coordinates of two protein structures, which has been proven an NP-hard problem [18]. To accelerate the alignment process, existing methods [6–10] generally apply heuristic algorithms. For example, heuristics are used in combinatorial extension (CE) for similarity evaluation and path extension [6]; Monte Carlo optimization is used in DALI for the assembly of alignments [7]; heuristic iteration combined with Needleman-Wunsch dynamic programming is used in STRUCTAL, SAL and TM-align to optimize the superposition [8–10,19].

Generally, existing alignment-based methods are time-consuming. When searching against a large-scale protein structure database, alignment-based methods would be infeasible. For instance, for *m* query structures and a database containing *n* structures, the time complexity of similar structure retrieval will be O(*mn*) if the database is not specifically designed. For example, the 2.07 version of Structural Classification of Proteins-extended (SCOPe) database contains 87,224 PDB entries and 276,231 domains [20,21]. TM-align, one of the popular alignment-based methods, takes about 0.5 sec for one structural alignment on a 1.26 GHz PIII processor [10]. In total, it will take about 138,115 secs (more than 38 hours) for a single query to retrieve all similar domains. In addition, recent protein structure prediction tools can predict protein structures from sequences with a remarkable accuracy. It is expected that these prediction algorithms will soon be applied to the protein sequences with unknown structures. For example, over 8 million protein sequences are currently deposited in the NR database, which would result in millions of predicted protein structures in a near future. If searching such a big number of predicted protein structures against the PDB database, the time cost of alignment-based 3D structure comparison (e.g. TM-align) will be unaffordable. Thus, it is an urgent task to develop more efficient protein structure comparison methods for large-scale protein structure homology retrieval due to the explosion of protein 3D structure data.

Compared with the alignment-based methods, alignment-free methods follow a different paradigm that represents a protein structure using a vector. This vector is named *descriptor* in this study by following the practice in other machine learning tasks [22,23]. In general, descriptors need to satisfy two requirements:1) the length should be fixed and independent of the size of proteins; 2) they should be invariant to rotation and translation of proteins.

Generally, the alignment-free methods can be divided into three groups according to their ways of obtaining the descriptors. The first group is the geometry-based method, which extracts predefined geometric features from protein structures. For example, the scaled Gauss metric (SGM) is proposed based on knot theory [14]. It treats the protein backbone as a space curve and extracts Gaussian invariants from the curve. The second group is the frequency-based method. It first splits the whole protein structure into many short consecutive segments and then classifies each segment into one predefined type. For example, the secondary structure element footprint (SSEF) method [15] uses the frequencies of the combination of secondary structures as descriptors. Fragbag constructs a fragment template library and each segment in the backbone is associated with the most similar template in the library [16]. Frequencies of each fragment will be counted to form the descriptors. Both groups of alignment-free methods rely on hand-crafted geometric features.

The third group is the learning-based method. Learning-based methods try to work around the sequence segmentation and feature engineering by learning structural representation automatically using deep neural networks. DeepFold is such a method, which extracts descriptors from intra-residue distance matrices under a Siamese framework with a convolutional neural network (CNN) encoder [24]. DeepFold model has suggested that deep learning techniques are able to extract more discriminative descriptors than the former two hand-designed alignment-free methods. However, DeepFold has a large number of parameters, which would decrease its efficiency. In addition, CNNs also could face challenges of effectively capturing the spatial relationships among residues in protein structures [25]. Alternatively, graph neural networks (GNNs) can be designed to handle the spatial graph data derived from protein structures with impressive performance [26].

In this paper, we propose a novel Graph-based protein Structure Representation (GraSR) method with deep GNNs. GraSR first represents the protein structures using graphs based on the intra-residue distance. Then, a contrastive learning framework is used to optimize the encoder, where TM-score derived from TM-align is used as the reference benchmark [27]. The encoder in GraSR consists of the long short-term memory neural network (LSTM) and GNN instead of the CNN [28,29]. Compared with CNN, GNN and LSTM have much fewer parameters, speeding up the training and inference procedure. In addition, GNN can learn global and local geometric features of residues better. Moreover, a new training data partition strategy and a length-based normalization technique are designed to further improve the performance. Several state-of-the-art methods and GraSR are evaluated on SCOPe v2.07 and an independent test set constructed from the newly released PDB. The results show that GraSR retrieves more similar protein structures and the extracted descriptors are more discriminative for fold recognition.

## Materials and methods

In this section, we first introduce the benchmark datasets for evaluating GraSR. Then, we give the details about graph construction from protein structures and the proposed GNN encoder, which is trained under the framework of contrastive learning with two novel training strategies. Finally, experimental settings for ranking and multi-class classification tasks are given in detail.

### Benchmark datasets

In this study, we use SCOPe v2.07 (March 2018) as the benchmark set. The 40% identity filtered subset of SCOPe v2.07 is used to train and validate our model GraSR. This dataset contains 14,323 domains and 1,058 domains are removed during the data collection process (cf. Text A in S1 File). Thus, 13,265 domains are finally used for cross-validation. Each domain can be classified into one of the seven classes [20] including: a) All alpha proteins (2286 domains), b) All beta proteins (2757 domains), c) Alpha and beta proteins (a/b) (4148 domains), d) Alpha and beta proteins (a+b) (3378 domains), e) Multi-domain proteins (alpha and beta) (279 domains), f) Membrane and cell surface proteins and peptides (213 domains), and g) Small proteins (204 domains).

In addition to SCOPe v2.07, we have also constructed an independent test set by using protein structures from PDB, the release date of which is from Oct 1^st^ 2017 to Oct 1^st^ 2019 (i.e., the date after the publication of DeepFold). If a certain PDB file contains multiple chains, it will be split into multiple files, each of which contains only one chain. Then, CD-HIT is used to remove the redundant sequences [30]. The sequence identity of the independent set itself and its sequence identity to SCOPe v2.07 are both below 40% after filtering. At last, 51 protein chains are removed due to the same technical issues as SCOPe. The final independent test set (named ind_PDB) contains 1,914 protein structures.

### Graph construction and raw node feature extraction

The graph of a protein structure G=(V,E) is constructed based on the Cartesian coordinates of *C*_*α*_ atoms, where *V* is the set of nodes, *E* is the set of edges. In this study, each *C*_*α*_ atom is considered as a node and edges are defined between any two *C*_*α*_ atoms, which means G is a complete graph. The intra-residue distance matrix is denoted as $D\in N^{N_{r}\times N_{r}}$, and *N*_*r*_ is the number of residues in the given protein. Then, the adjacency matrix A is derived from D as following:

$$A_{ij}=\frac{\omega }{max(D_{ij},\epsilon )}$$ (1)

where *ω* and *ϵ* are two hyperparameters for normalization and $D_{ij}$ is the Euclidean distance between the *i*^*th*^ *C*_*α*_ atom and the *j*^*th*^ *C*_*α*_ atom. *ϵ* can also help avoid numerical error when $D_{ij}=0Å.\;A_{ij}$ is normalized to (0, 2] by setting *ω* = 4 and *ϵ* = 2.

**Fig 1:**
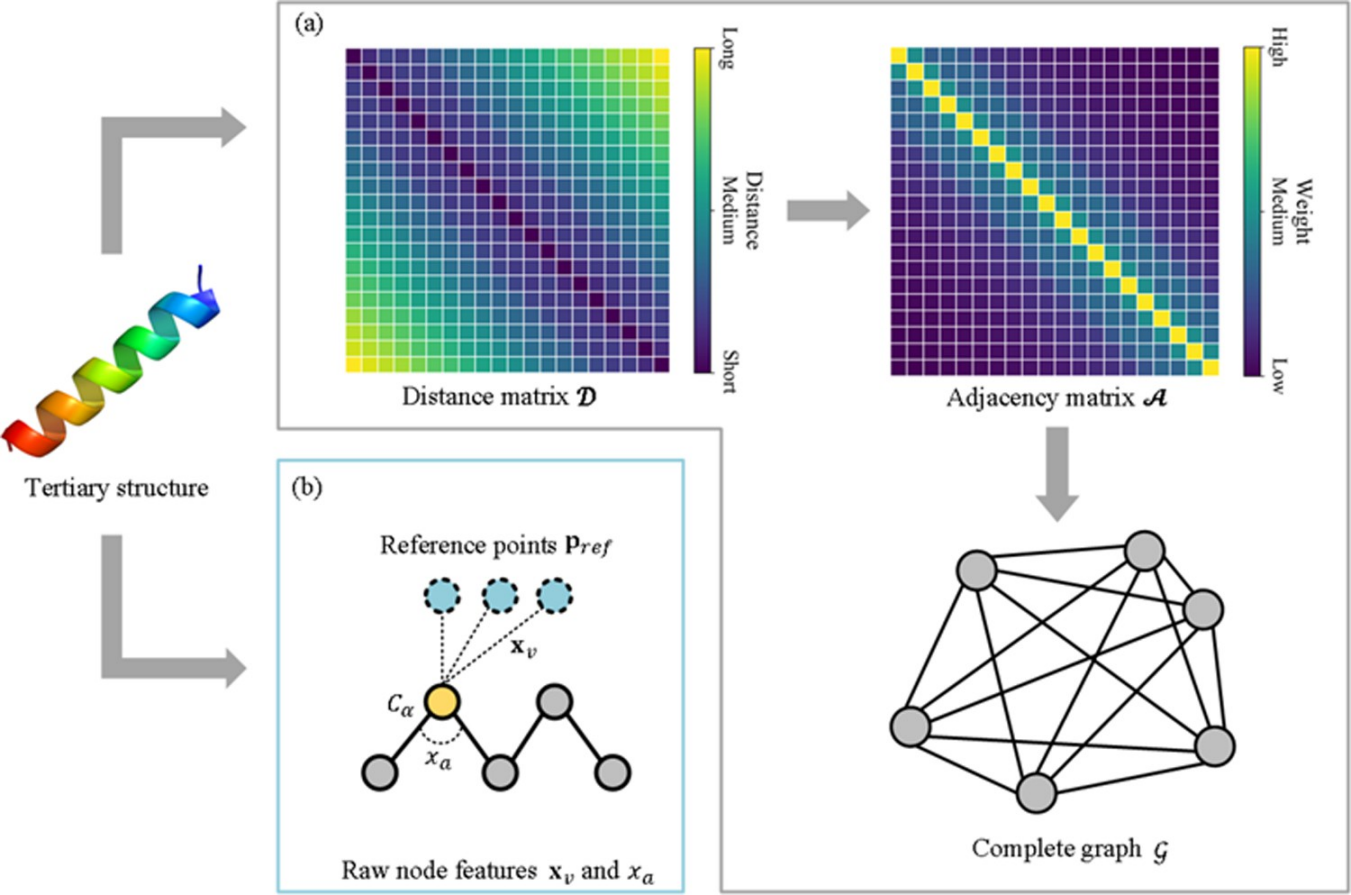
(A) The complete graph G is constructed based on protein tertiary structure, where the adjacency matrix is derived from the intra-residue distance matrix. (B) Raw node features consist of distance-based feature **x**_*v*_ and angle-based feature *x*_*a*_.

As shown in Fig 1B, two types of raw node features are extracted, and they are invariant to rotation and translation. One is distance-based features, which is derived from the distance between the target residue and some specific points in the three-dimensional space. The coordinate of one of these points is calculated as following:

$$p_{ref}=\frac{1}{j-i}\sum_{v\in V_{i:j}}v$$ (2)

where $V_{i:j}=\{v_{i},v_{i+1},\ldots ,v_{j-2},v_{j-1}\}(i<j)$ and **v**_*i*_ denotes the Cartesian coordinate of *i*^*th*^ residue in the protein sequence. These points are named reference points in this study. The coordinates of all reference points and the raw node features of the target residue can be derived as Algorithm 1. The length of the distance-based raw node feature vector **x**_*v*_ is 2^*M*^−1, where *M* is a hyperparameter controlling the number of reference points. A proof that the distance-based feature is invariant to rotation and translation can be found in Text D of S1 File

**Algorithm 1**. Distance-based raw node feature extraction from the protein structure graph.

**Input** The set of nodes: $V=\{v_{1},v_{2},\ldots ,v_{N_{r}-1},v_{N_{r}}\}$

        The number of reference points: 2^*M*^−1(*M*∈ℕ)

        The coordinate of the target residue: **v**_*tgt*_∈*V*

**Output** The distance-based raw node feature vector of a target residue: **x**_*v*_

**Step** t = 0

        # Reference points are divided into *M* groups. The *m*^*th*^ group consists of 2^*m*^ points.

        for *m* in {0,1,…,*M*−1}:

            # Calculate the *g*^*th*^ reference point in the *m*^*th*^ group.

            for *g* in {1,2,3,…,2^*m*^}:

                # The subset $V_{\left\lfloor (g-1)\frac{N_{r}}{2^{m}}\rfloor :\lceil g\frac{N_{r}}{2^{m}}\right\rceil }$ refers to a continuous fragment in a protein.

            $p_{ref}^{\left(t\right)}=\frac{2^{m}}{N_{r}}\sum_{v\in V_{\left\lfloor (g-1)\frac{N_{r}}{2^{m}}\rfloor :\lceil g\frac{N_{r}}{2^{m}}\right\rceil }}v$

                t++

        $x_{v}={\left\{\left\|v_{tgt}-p_{ref}^{\left(t\right)}\right\|\right\}}^{2^{M}-1}\;(t=0,1,\ldots ,2^{M}-2)$

The other is angle-based features, which are derived from the angles formed by the *C*_*α*_ atoms of three consecutive residues in the structure and can be calculated as following:

$$x_{a}=\frac{{\left({v_{i+1}-v}_{i}\right)}^{T}({v_{i+1}-v}_{i})}{\left\|{v_{i}-v}_{i-1}\|\|{v_{i+1}-v}_{i}\right\|}$$ (3)

where *x*_*a*_ denotes the angle-based raw feature of the *i*^*th*^ residue in the protein sequence. When *i* = 1 or *N*_*r*_, *x*_*a*_ = 0. The final raw node features with a length of *d* = 2^*M*^ concatenate the distance-based and angle-based features.

### GNN-based encoder

The graph convolutional neural network (GCN) is motivated by the spectral graph theory and is proposed for learning on graph-structured data [28,31]. To date, many graph neural networks (GNNs) are proposed to extend the GCN, such as the message passing neural network (MPNN) and GraphSage [32,33]. Most of GNNs are designed to update the node embeddings by aggregating the information from their neighboring nodes.

In this study, a graph convolutional layer is designed to learn the geometric features of each residue and its neighborhood in the three-dimensional space as following:

$$X^{l+1}=\sigma (AX^{l}W^{l})$$ (4)

where $X^{l}\in R^{N_{r}\times d}$ is the node feature matrix of the *l*^*th*^ layer, **W**^*l*^∈ℝ^*d*×*d*^ is the learnable weight matrix of the *l*^*th*^ layer, A is the adjacency matrix, and σ(∙) is a nonlinear activation function.

Eq 4 can also be written as:

$$x_{j}^{l+1}=\sigma (\sum_{k=1}^{N_{r}}a_{jk}x_{k}^{l}∙W^{l})$$ (5)

where $x_{j}^{l+1}$ is the node embeddings of the *j*^*th*^ node in the graph (i.e., the *j*^*th*^ row of **X**^*l*^) and *a*_*jk*_ is the element of A.

It is obvious that the central node feature is affected more by the nodes close to it instead of distant ones according to the Eq 5. The adjacency matrix plays the role of a weight matrix. Here we do not normalize the adjacency matrix since self-loop and normalization are already applied to the adjacency matrix during graph construction.

Multiple graph convolutional layers are stacked to learn high-level geometric features in our model. To overcome the gradient-vanishing and over-smoothing problem, residual blocks are built by adding identity shortcuts [34]. Each residual block contains two graph convolutional layers and can be defined as following:

$$X^{l+2}=\sigma (A{X^{l+1}W}^{l+1}+X^{l}W_{s})$$ (6)

where **W**_*s*_ is used to match the dimension when the sizes of **X**^*l*+1^ and **X**^*l*^ are different.

The primary structure of a protein consists of a sequence of amino acids (nodes). However, GCN cannot capture the order of nodes because aggregator is invariant to permutation. Thus, before applying graph convolution, a bidirectional long short-term memory (BiLSTM) network is used to extract low-level features from sequential context [35]. BiLSTM consists of two LSTMs. Each of them takes the same protein sequence as the input but from different directions. The hidden state at each time step of these two LSTMs is concatenated as the initial node embeddings for the following graph convolutional layers.

**Fig 2:**
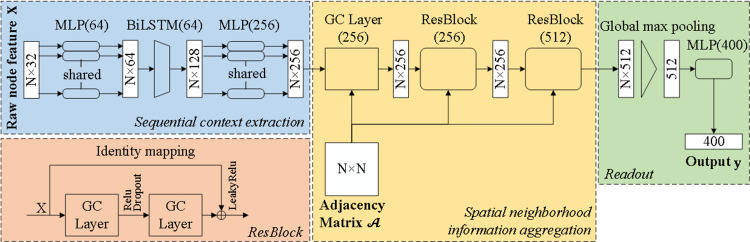
Architecture of GNN-based encoder. The BiLSTM module extracts low-level node features from the primary structures of proteins. The graph convolution module extracts high-level node features based on the adjacency matrices A. The readout module transforms node features to the descriptors by a global max pooling layer. The residual blocks (ResBlock) used in the graph convolutional module consists of two graph convolutional (GC) layers.

As shown in Fig 2, the GNN-based encoder consists of three modules: the first module is used to extract sequential context, which consists of two multi-layer perceptrons (MLPs) and a BiLSTM network; the second module consists of multiple graph convolutional layers; in the last module, a global max pooling layer is used to summarize the final graph embeddings.

### The contrastive learning framework

A contrastive learning framework usually consists of multiple neural networks sharing the same architecture and parameters. These neural networks serve as encoders and each of them transforms a sample to the corresponding descriptor. The loss function is dependent on the distance between these descriptors. If samples are similar, the distance should be minimized; otherwise, the distance should be maximized.

**Fig 3:**
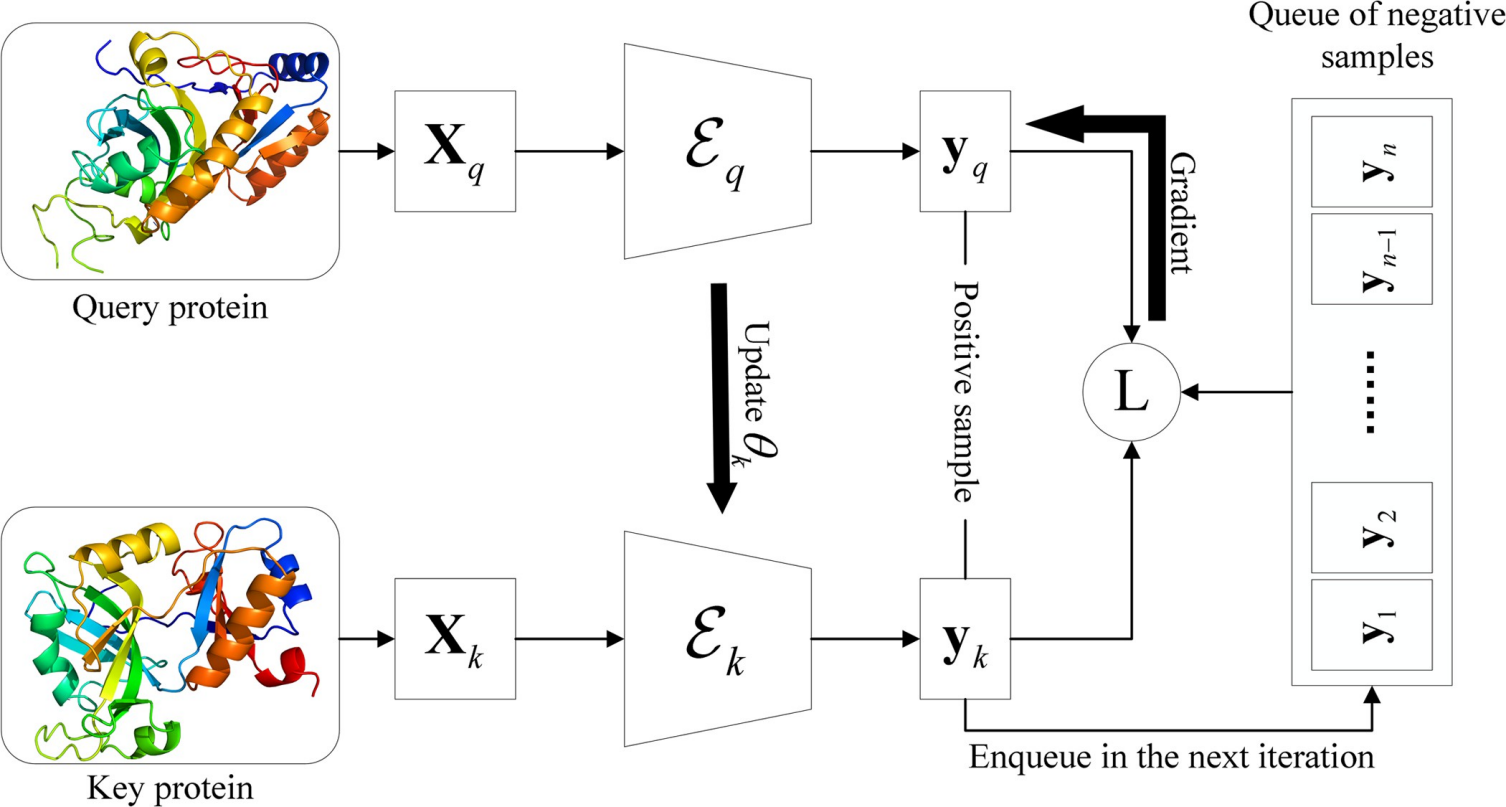
The contrastive learning framework for protein structure representation learning. At each iteration, raw features **X**_*q*_ and **X**_*k*_ are extracted from the query protein structure and the key protein structure, respectively. Then, descriptors **y**_*q*_ and **y**_*k*_ are encoded by GNN encoder $E_{q}$ and $E_{k}$, respectively. The value of loss function guides the optimization of the parameters *θ*_*q*_ of $E_{q}$ while the parameters *θ*_*k*_ are updated based on *θ*_*q*_. At the end of the current iteration, **y**_*k*_ will enqueue as a negative sample for the next iteration.

In GraSR, Momentum Contrast (MoCo) is used as a contrastive learning framework, which was originally proposed for unsupervised visual representation learning [36]. We apply it to protein structure representation by substituting its CNN encoders with our GNN encoders. As shown in Fig 3, MoCo consists of two GNN-based encoders $E_{q}$ and $E_{k}$, which share the same architecture. However, these two encoders have different parameter sets of *θ*_*q*_ and *θ*_*k*_. The parameter set *θ*_*q*_ is updated by back-propagation. The parameter set *θ*_*k*_ is updated by *θ*_*q*_ as following:

$$\theta_{k}\leftarrow m∙\theta_{k}+(1-m)∙\theta_{q}$$ (7)

where *m*∈(0, 1] is a momentum coefficient.

The input of two encoders are raw node features $X_{q}\in R^{l_{q}\times d}$ and $X_{k}\in R^{l_{k}\times d}$ extracted from the query protein structure and the key protein (i.e., the protein in the database) structure, respectively. *l*_*q*_ is the number of residues in the query protein, *l*_*k*_ is the number of residues in the key protein, and *d* is the dimension of raw features. When constructing the training data, we guarantee that the key protein structure is the structural neighbor (i.e., similar structure) of the query protein in the current mini-batch and thus it can be seen as a positive sample. Moreover, the chosen query protein must be dissimilar to previous *n* key proteins. The outputs of MoCo are two descriptors **y**_*q*_∈ℝ^*L*^ and **y**_*k*_∈ℝ^*L*^, where *L* is the length of the descriptors. At the end of each iteration, **y**_*k*_ is pushed into a randomly initialized queue of length *n* during training. All descriptors in the queue can be used as the negative samples (i.e., dissimilar structures) in the next iteration due to the specific data construction. If the queue is full, the earliest samples dequeue.

The loss function called InfoNCE [37] is used to optimize the encoder $E_{q}$ as following:

$$L=-\log\frac{e^{y_{q}∙\frac{y_{k}}{\tau }}}{\sum_{i=1}^{n}e^{y_{q}∙\frac{y_{i}}{\tau }}}$$ (8)

where τ is a temperature hyperparameter, *n* is the length of the queue, and **y**_*i*_ is the descriptor of *i*^*th*^ negative sample in the queue. Dot-product is used to measure the cosine similarity between **y**_*q*_ and other descriptors because **y**_*q*_, **y**_*k*_ and **y**_*i*_ are normalized to 1.

### Length-scaling cosine distance

In the training stage, cosine similarity is used to measure the similarity between two descriptors. Cosine similarity is symmetric, which means cos(**y**_*a*_, **y**_*b*_) = cos(**y**_*b*_, **y**_*a*_). However, TM-score is dependent on the length of protein sequences and thus asymmetric according to its definition [27]. Length-scaling cosine distance is proposed to bridge the gap between cosine similarity and TM-score. Cosine distance is defined as $1-\cos\left(y_{a},y_{b}\right)=1-y_{a}^{T}y_{b}/\left\|y_{a}\|\|y_{b}\right\|$. It can be rewritten as $1-y_{a}^{T}y_{b}$ if ‖**y**_*a*_‖ = ‖**y**_*b*_‖ = 1. Thus, we define the length-scaling cosine distance as following:

$$d(y_{a},y_{b})=\frac{1-y_{a}^{T}y_{b}}{1+\max\left(\frac{l_{b}-l_{a}}{l_{max}},0\right)}\in [0,2]$$ (9)

where *l*_*a*_, *l*_*b*_, and *l*_*max*_ denote the length of query sequence A, key sequence B and the longest sequence in the database. When *l*_*b*_<*l*_*a*_, it is equivalent to standard cosine distance; otherwise, the distance between **y**_*a*_ and **y**_*b*_ will be decreased. d(**y**_*a*_, **y**_*b*_)>d(**y**_*b*_, **y**_*a*_) if *l*_*a*_<*l*_*b*_, which is more consistent with TM-score.

Length-scaling cosine distance is only used in the testing stage for ranking. In the training stage, cosine similarity is still applied to model optimization.

### Dynamic training data partition

In the previous learning-based method DeepFold, it uses the following data partition strategy: for each sample pair (**X**_*a*_, **X**_*b*_), if its TM-score is higher than *ρ*∙TM_*max*_(X_*a*_), **X**_*b*_ is seen as a positive sample to **X**_*a*_, where TM_*max*_(X_*a*_) is the maximal TM-score between **X**_*a*_ and other protein structures in the target database [24]. *ρ*∈(0,1) is a hyperparameter and is set to 0.9. The above strategy for training data partition used in DeepFold is the same as that used for test data partition. It means that the model only needs to learn TM(**X**_*q*_, **X**_P_)>TM(**X**_*q*_, **X**_N_), where **X**_P_ is any positive sample to **X**_*q*_ and **X**_N_ is any negative sample to **X**_*q*_. The relationship between any positive pairs and the relationship between any negative pairs remain unknown to the model.

To resolve the above issue, we design a dynamic training data partition strategy. At first, for each query structure, all structures in the database are sorted according to their TM-score. Then, the Top *K* percent (e.g., 30%) structures are used to construct a subset, which is denoted as S. At each iteration, a structure is randomly sampled fromS as a positive sample **X**_S_. Any structures in the database (no matter whether it is in S) with TM-score less than TM(**X**_*q*_, **X**_S_) are seen as negative samples. If the neural network is trained for infinite iterations, each structure in S will be sampled at least once. Therefore, the relationship among all samples in the S can be learned by the neural network using the dynamic training data partition strategy.

## Experimental settings

### Evaluation protocols

The 5-fold cross-validation is conducted to train and validate GraSR on SCOPe v2.07. Other methods for comparison are tested by running their standalone software. To comprehensively evaluate the performance, each method is evaluated for a ranking task and a classification task.

In the ranking task, all methods are also evaluated on the independent test set ind_PDB. For each query, each method ranks all structures in the database in descending/ascending order according to the similarities/distance. The ranking result is compared against the result of TM-align.

To make a fair comparison, similar settings with the latest method, DeepFold, are used [24]. For each query structure in test set, the structures with TM-score no less than 0.9*TM-score_max (the highest TM-score in the training set) are considered as structural neighbors, namely positive samples.

Similar to DeepFold and Fragbag, we calculate the area under Receiver operating characteristics (AUROC) curves and the area under precision-recall curves (AUPRC) as performance metrics. However, AUROC would overestimate the performance when data is severely imbalanced [38]. Considering that protein structures generally have very few structural neighbors, ROC may not be a reliable metric here. Thus, we are more focused on AUPRC when comparing different methods.

In addition, Top-K accuracy could also not be comprehensive. For example, two algorithms find one and two structural neighbors in Top-10, respectively. The latter performs better. However, both are considered as hits according to the definition of Top-K accuracy. To avoid the problem, we propose a new metric named Top-K hit ratio based on Top-K accuracy as following:

$${Ratio}_{K}=\frac{1}{N_{q}}\sum_{i=1}^{N_{q}}\frac{N_{hit}^{i}}{\min\left(K,N_{nbr}^{i}\right)}$$ (10)

where $N_{hit}^{i}$ denotes the number of structural neighbors found by the algorithm for the *i*^*th*^ query, $N_{nbr}^{i}$ denotes the total number of structural neighbors for the *i*^*th*^ query, and *N*_*q*_ denotes the number of queries. This metric can be seen as an extension to Top-K accuracy. When *K* = 1, Top-K hit ratio is equivalent to Top-K accuracy.

In summary, AUROC, AUPRC, Top-1 hit ratio, Top-5 hit ratio, and Top-10 hit ratio are selected to evaluate all methods in the ranking task. AUROC/AUPRC is calculated for each query, and the average of AUROC/AUPRC is used to evaluate the overall performance on the whole dataset.

Each domain in SCOPe v2.07 belongs to a specific class. In the classification task, all methods are used to generate descriptors from domains. Then, logistic regression (LR) classifiers are trained and used to predict the class of descriptors using 10-fold cross-validation. Considering it is a multi-class classification problem, average F1-score and accuracy are used as the evaluation metrics instead of ROC or PRC.

In addition, statistical hypothesis tests are further conducted to verify whether the performance difference between GraSR and compared methods is significant by following the protocol used in [39]. Half of the proteins in the validation or test set will be sampled randomly. The predictive performance of all methods will be evaluated on this subset. The procedure will be repeated 10 times and then GraSR will be compared with other methods on the 10 pairs of results. Paired t-test or Wilcoxon signed-rank test will be applied, which depends on whether the measurement follows a Gaussian distribution.

### Parameter setting details

The hyperparameter *M* is set to 5, which means the number of reference points is 31. Following the practice in the original paper [36], in the MoCo framework, the momentum *m* is set to 0.999 and the temperature τ is set to 0.07. The length of the queue of negative samples is set to 1024. When applying the dynamic training strategy, Top-30% structures are used to construct the subset S. The length of the raw node feature vector *d* is set to 32. Stochastic gradient descent (SGD) is used to optimize the neural networks and the momentum of SGD is set to 0.9. The size of mini-batch is set to 64. Initial learning rate is set to 0.1 and divided by 10 when AUPRC plateaus. Each model during cross-validation is trained for up to 2.4×10^5^ iterations. Shuffled batch normalization (BN) is used after each layer [40]. Rectified linear unit (ReLU) and dropout is used in the Res Block after the first GC layer [41,42]. Leaky ReLU is used in the other layers [43]. The whole GraSR model is trained on two TITAN Xp Graphics Cards, and the training procedure took several days.

## Results

In this section, we first perform ablation studies to evaluate the effectiveness of two proposed training strategies and GNN-based encoder in GraSR. Then, we compare GraSR with baseline methods for ranking and multi-classification task on two benchmarked datasets. Finally, we also evaluate the computational efficiency of GraSR and baseline methods.

### Ablation studies

In this section, we evaluate the effectiveness of the length-scaling cosine distance, the dynamic training data partition strategy, the raw node features, the BiLSTM layer and the GNN-based encoder, which are designed for GraSR. All experiments are performed on the SCOPe v2.07 and ind_PDB. Six variants of GraSR are used to compare:

- w/o LS: length-scaling cosine distance used in GraSR is substituted with standard cosine distance.
- w/o DP: dynamic training data partition strategy is not used.
- w/o RNN: remove the BiLSTM layer from the encoder.
- GraSR-dist: Only the distance-based raw node feature is used.
- GraSR-angle: only the angle-based raw node feature is used.
- GraSR-CNN: the GNN-based encoder used in GraSR is substituted with a CNN-based encoder. The architecture of the CNN is similar to the one used in DeepFold [24] (cf. Text B in S1 File).

The results in Table 1 show that the performance degrades in general if any component of GraSR is removed or substituted of these variants in our local tests. Avg. AUPRC, Top-1 hit ratio, Top-5 hit ratio and Top-10 hit ratio of GraSR increase 10.01%/6.86%, 10.34%/7.63%, 9.86%/7.11% and 8.74%/5.85% respectively when compared with GraSR-CNN. These results demonstrate that our proposed GNN-based encoder is more suitable for protein structure representation than the common CNN. The BiLSTM layer is also indispensable because the variant achieves the worst performance when it is removed. In addition, the dynamic training data partition strategy obtains ~1–2% increase on the four metrics, respectively. It proves that even if no change is made to the main algorithm architecture, refining the data labelling is able to improve its performance. The improvement brought by length-scaling cosine distance is also significant except the Top-10 hit ratio on the ind_PDB. The result of GraSR-dist and GraSR-angle shows that distance-based node features are more important while angle-based node features can help improve the Avg. AUROC. The overall results show that all introduced components contribute to the superior performance of GraSR.

**Table 1:** Ablation studies of length-scaling cosine distance, the dynamic training data partition strategy and the GNN-based encoder on SCOPe v2.07 and ind_PDB.

Dataset	Method	Avg. AUROC^†^	Avg. AUPRC^†^	Top-1^†^	Top-5^†^	Top-10^†^
SCOPe v2.07	w/o LS	0.9752**	0.6521*	0.7188**	0.7063*	0.7370*
	w/o DP	0.9743***	0.6426**	0.7038**	0.7007**	0.7275**
	w/o RNN	0.9603***	0.5005***	0.5630***	0.5541***	0.5954***
	GraSR-dist	0.9816*	0.6587	0.7260	0.7104	0.7406
	GraSR-angle	0.9803**	0.6073***	0.6674***	0.6650***	0.7042***
	GraSR-CNN	0.9781**	0.5594***	0.6248***	0.6115***	0.6526***
	GraSR	0.9823	0.6595	0.7282	0.7101	0.7400
ind_PDB	w/o LS	0.9436**	0.3919*	0.4383*	0.4388*	0.4698
	w/o DP	0.9382**	0.3881*	0.4333*	0.4370*	0.4665
	w/o RNN	0.8999***	0.2640***	0.3052***	0.3019***	0.3224***
	GraSR-dist	0.9539	0.4016	0.4483	0.4462	0.4818
	GraSR-angle	0.9615*	0.3670**	0.4085**	0.4098**	0.4391**
	GraSR-CNN	0.9623**	0.3372**	0.3795**	0.3777**	0.4179**
	GraSR	0.9528	0.4058	0.4558	0.4488	0.4764

*p-value of t-test is < 0.05

**p-value of t-test is < 10^−4^

***p-value of t-test is < 10^−9^.

^†^Avg. AUROC, Avg. AUPRC and Top-K hit ratio are in [0, 1], the bigger the better.

### Comparing GraSR with the state-of-the-art alignment-free methods

GraSR is compared with three state-of-the-art structure representation methods: SGM, SSEF and DeepFold [14,15,24]. All methods are evaluated for the ranking task and classification task on two benchmark datasets. Moreover, the computational efficiency of structure representation methods is also benchmarked.

### GraSR is superior to baseline methods on the ranking task

The results summarized in the Table 2 show that GraSR significantly outperforms other baseline methods. Another deep-learning-based method DeepFold achieves the second best performance. Compared with DeepFold, GraSR achieves about 10%/7% improvement on SCOPe v2.07/ind_PDB. The results indicate that the architecture of our proposed GNN is superior to CNN of DeepFold on this task. In addition, it can be observed that two deep-learning-based methods perform better than other methods based on hand-crafted descriptors. This observation demonstrates that the descriptors automatically learned from large-scale data reserve more structural information. DeepFold, SGM and GraSR perform better on SCOPe v2.07 than on ind_PDB. The reason is that these methods are trained or designed based on SCOPe or CATH, each entry of which represents a single domain. However, on the ind_PDB, each structure may contain multiple domains. Even so, GraSR still yields an AUPRC of over 0.4, which is higher than that of other baseline methods.

**Table 2:** Ranking performance of GraSR and other baseline methods.

Dataset	Method	Avg. AUROC^†^	Avg. AUPRC^†^	Top-1^†^	Top-5^†^	Top-10^†^
SCOPe v2.07	SGM	0.9224**	0.4537**	0.5562**	0.5312**	0.5553**
	SSEF	0.8423**	0.0381**	0.0838**	0.0580**	0.0610**
	DeepFold	0.9574**	0.4971**	0.6035**	0.5659**	0.5927**
	GraSR	0.9823	0.6595	0.7282	0.7101	0.7400
ind_PDB	SGM	0.8167**	0.2231**	0.2745**	0.2681**	0.2850**
	SSEF	0.8281**	0.0433**	0.0474**	0.0400**	0.0460**
	DeepFold	0.9339*	0.3144**	0.3819*	0.3662*	0.3916*
	GraSR	0.9528	0.4058	0.4558	0.4488	0.4764

*p-value of t-test is < 10^−4^

**p-value of t-test is < 10^−9^.

^†^Avg. AUROC, Avg. AUPRC and Top-K hit ratio are in [0, 1], the bigger the better.

**Fig 4:**
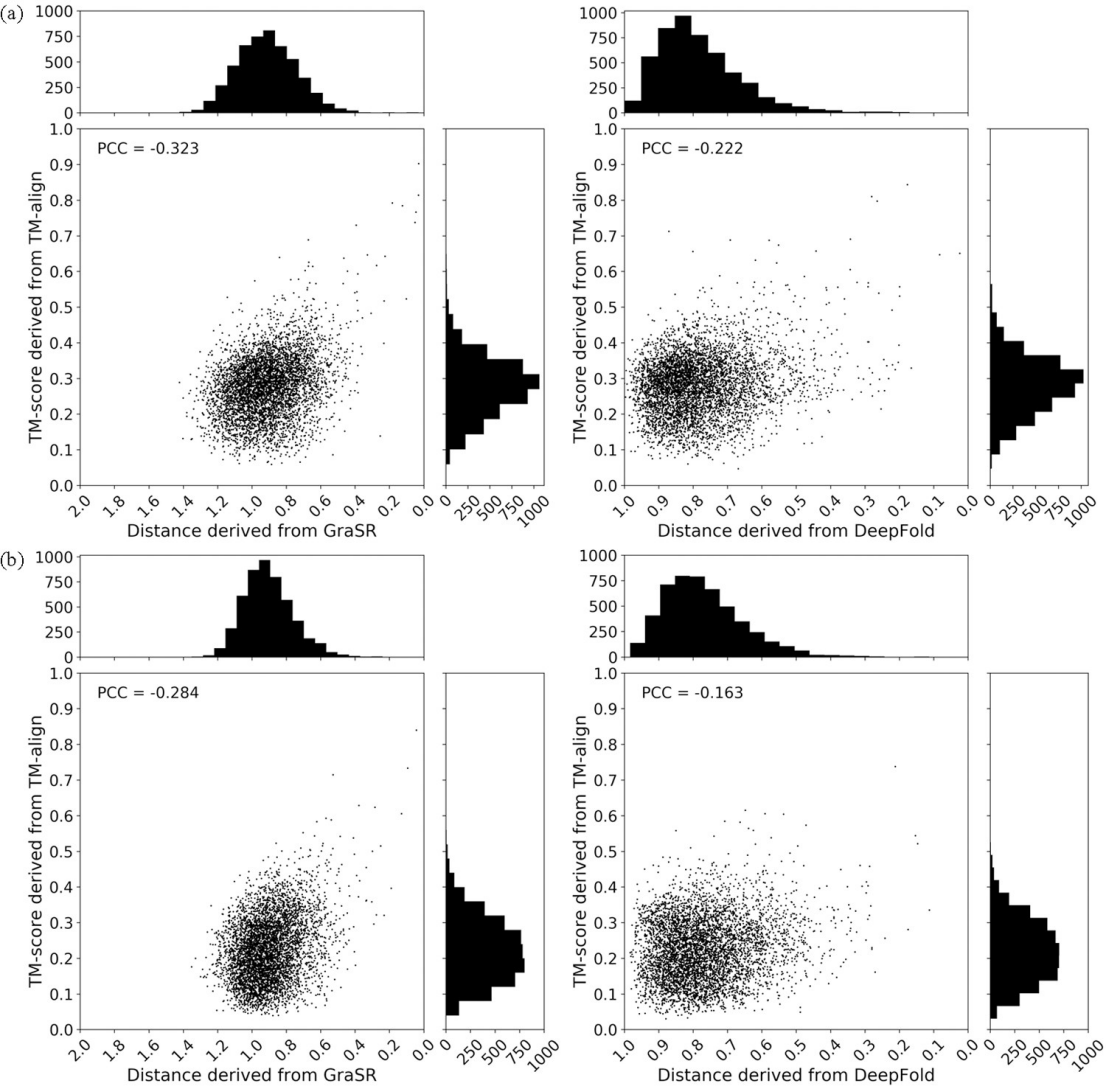
Correlation between distance derived from the representations learned by GraSR/DeepFold and TM-score on (A) SCOPe v2.07 and (B) ind_PDB. The Pearson correlation coefficient (PCC) is calculated for quantitative assessment.

Considering that DeepFold is also trained using the labels derived from TM-score, we further compare GraSR with it. We sample 5,000 structure pairs randomly from SCOPe v2.07 and ind_PDB, respectively. The correlation between the distance derived from the representations learned by GraSR/DeepFold and TM-score of these structure pairs are shown in Fig 4. The Pearson correlation coefficient (PCC) of both methods are smaller than zero, and the negative correlation between the distance and TM-score is expected. The |PCC| (absolute value of PCC) of GraSR is 10.1%/12.1% higher than that of DeepFold on SCOPe v2.07/ind_PDB. The results demonstrate that the similarity between two descriptors derived from GraSR is more correlated to the TM-score, which is derived from an alignment-based method TM-align. The PCC of both methods are not very high because they focus on the structure pairs with relatively high TM-score. If we remove the structure pairs with TM-score smaller than 0.5, the |PCC| of GraSR will increase to 0.600/0.556 on SCOPe v2.07/ind_PDB. The high correlation to TM-score elucidates the effectiveness of GraSR.

### GraSR outperforms baseline methods on the classification task

GraSR and other structure representation methods are further evaluated by predicting the classes of proteins in SCOPe v2.07. There are totally 7 classes in the 40% identity filtered subset of SCOPe v2.07 [20]. We first use GraSR and other baseline methods to learn the structure representations, which are then fed into a multi-class LR for fold recognition.

**Fig 5:**
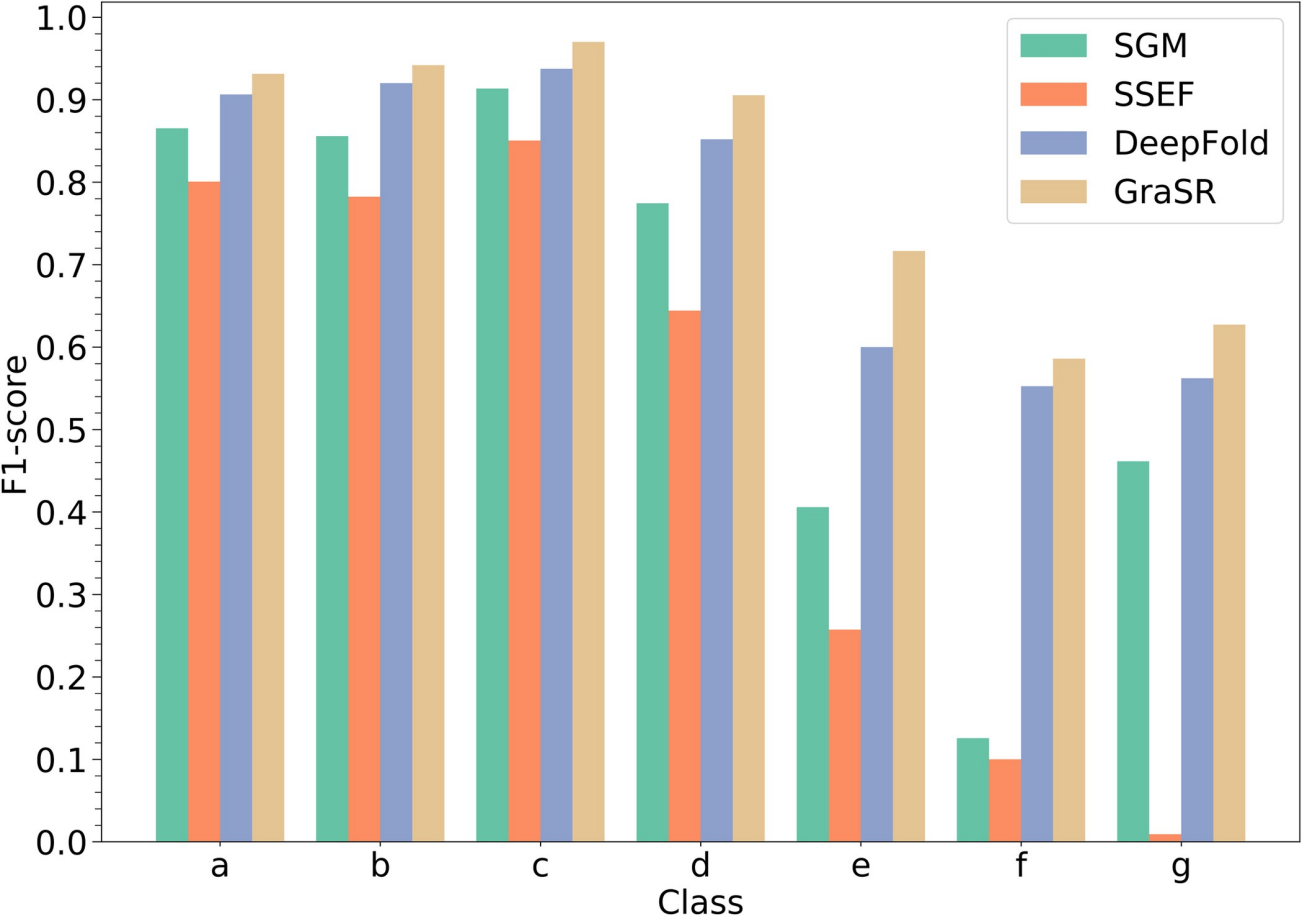
The F1-score of each class in SCOPe of GraSR and other baseline methods. a: All alpha proteins; b: All beta proteins; c: Alpha and beta proteins (a/b); d: Alpha and beta proteins (a+b); e: Multi-domain proteins (alpha and beta); f: Membrane and cell surface proteins and peptides; g: Small proteins.

The results are summarized in the Table 3. It can be observed that the results of the classification task are consistent with those of the ranking task. GraSR achieves the highest average F1-score and accuracy, followed by CNN-based DeepFold. Compared with DeepFold, GraSR further increases the average F1-score/accuracy by 5.1%/3.7%. The improvement is significant according to the *p*-value of t-test. Considering that LR is a linear classifier, the improvement is mainly owing to the learned discriminative descriptors. In addition, GraSR yields higher F1-scores on all seven classes than those of DeepFold (cf. Fig 5).

**Table 3:** Multi-class classification performance of GraSR and other methods.

Method	Avg. F1-score	Accuracy
SGM	0.6289**	0.8354**
SSEF	0.4920**	0.7470**
DeepFold	0.7615*	0.8887**
GraSR	0.8124	0.9258

*p-value of t-test is < 10^−4^

**p-value of t-test is < 10^−9^.

As shown in Fig 5, the performance of SGM, DeepFold and our method is comparable on the all alpha proteins, all beta proteins, alpha and beta proteins (a/b) and alpha and beta proteins (a+b). However, two deep learning-based methods, GraSR and DeepFold, perform better than two hand-crafted descriptors, SGM and SSEF, for multi-domain proteins, membrane and cell surface proteins and peptides, and small proteins. We notice that the three classes contain relatively fewer domains. A potential reason is that deep learning methods can capture more complicated and detailed structural patterns that have not been fully explored.

### GraSR is computationally efficient

To evaluate the computational efficiency, we test the abovementioned alignment-free methods and one of the latest method, Geometricus [44], as following:

1. Record the time *t*_*q*_ for generating the descriptors of all query structures.
2. Record the time *t*_*k*_ for generating the descriptors of all key structures (i.e., the structures in the database).
3. Record the sum of time *t*_*d*_ for calculating the distance between each query structure and key structure.

All 1,914 protein structures in the dataset ind_PDB are treated as the query structures and key structures simultaneously. Therefore, the pairwise distance is calculated 1,914×1,914 = 3,663,396 times. The total time for structure retrieval can be calculated as *T*_*total*_ = *t*_*q*_+*t*_*k*_+*t*_*d*_. It can be simplified to *T*_*total*_ = 2*t*_*q*_+*t*_*d*_ since query structures and key structures are the same. In fact, the structures in databases are known, and the descriptors of them can be precomputed. Thus, the total time with precomputation is shown and denoted as $T_{total}^{\prime }=t_{q}+t_{d}$.

Only one logical core of Intel Xeon CPU E5-2630 v4 is used to run the programs of these methods. The results are summarized in the Table 4. Geometricus is the fastest among all alignment-free methods. Two deep learning methods are relatively slower due to a large number of parameters in the model. However, the time consumption is still acceptable and GraSR ($T_{avg}^{\prime }=1.75\;sec$) is much faster than DeepFold ($T_{avg}^{\prime }=4.69\;sec$) due to less parameters in GNN-based encoders of GraSR. Moreover, GraSR yields much better performance on ranking and classification task than SGM. Considering that deep learning methods can be accelerated easily by GPU, the gap between them can be reduced. In addition, the time consumption of alignment-based TM-align is also shown for comparison. There is a large gap between TM-align and alignment-free methods since finding the optimal superposition is time-consuming. The results demonstrate that alignment-free methods are still the fastest ways for protein structure retrieval from a large structure database. Our results suggest that GraSR is a promising choice when making the tradeoff between the accuracy and running time.

**Table 4:** Time cost of GraSR and other methods for protein structure retrieval from ind_PDB.

Method	*T*_*total*_^a^ (sec)	*T*_*avg*_^b^ (sec)	$T_{total}^{\prime }$^c^ (sec)	$T_{avg}^{\prime }$^d^ (sec)
SGM	950.87	0.50	512.84	0.27
SSEF	2222.77	1.16	1313.01	0.69
DeepFold	17753.80	9.28	8980.56	4.69
Geometricus^e^	447.55	0.23		
GraSR	6534.55	3.41	3355.81	1.75
TM-align	673833.95	352.06		

^a^Total time for protein structure retrieval from ind_PDB without precomputation.

^b^*T*_*avg*_ = *T*_*total*_/1914.

^c^Total time for protein structure retrieval from ind_PDB with precomputation.

^d^ $T_{avg}^{\prime }=T_{total}^{\prime }/1914$

^e^The length of the descriptors generated by Geometricus is dependent on the query structures. Thus, the descriptors of the structures in the database cannot be precomputed.

## Discussions

### GraSR learns discriminative structure representations

**Fig 6:**
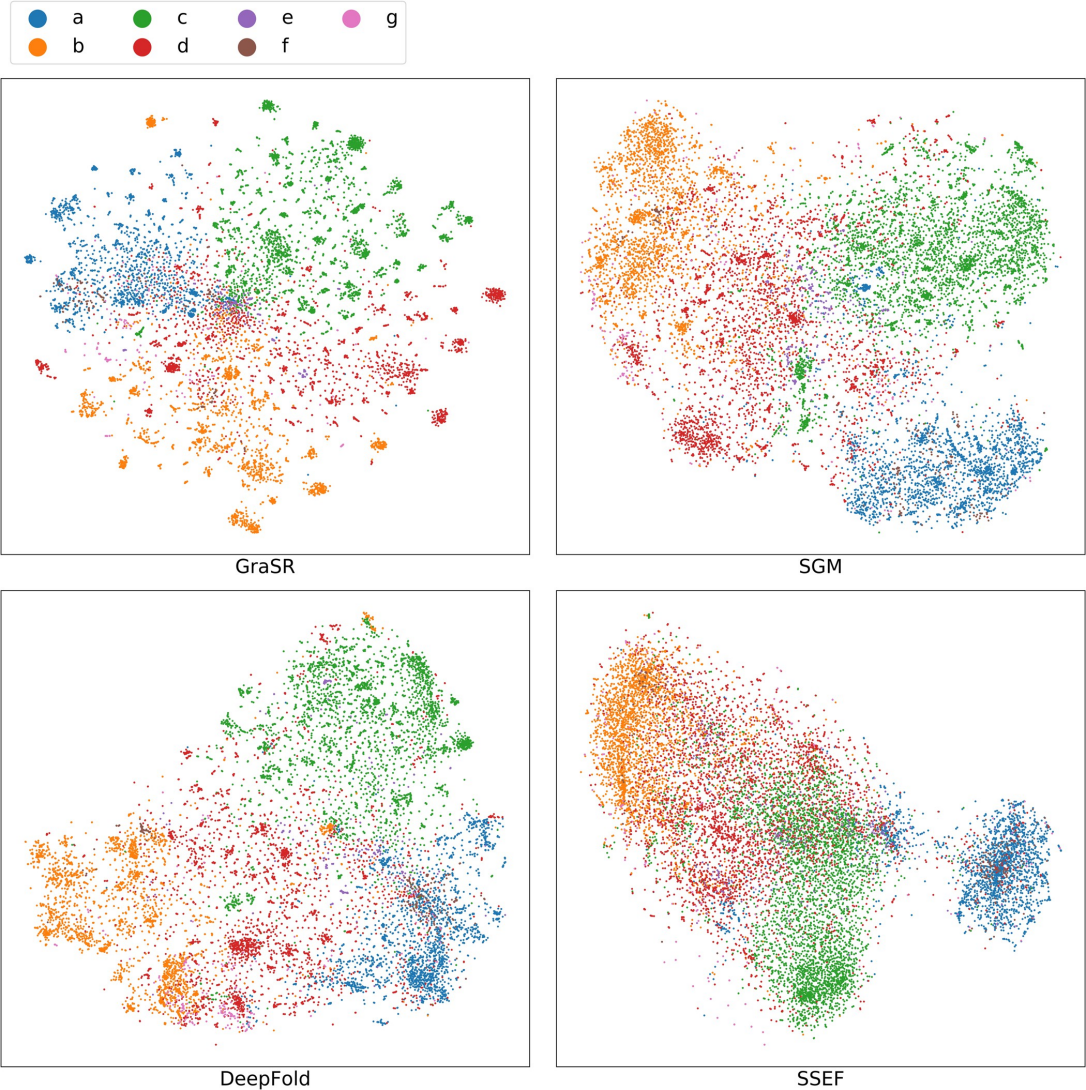
Visualization of descriptors learned from GraSR and other methods by t-SNE. a: All alpha proteins; b: All beta proteins; c: Alpha and beta proteins (a/b); d: Alpha and beta proteins (a+b); e: Multi-domain proteins (alpha and beta); f: Membrane and cell surface proteins and peptides; g: Small proteins.

To demonstrate whether the learned descriptors are discriminative, the descriptors generated by SGM, SSEF, DeepFold, and GraSR are visualized using t-distributed stochastic neighbor embedding (t-SNE) in Fig 6 [45]. The perplexity of t-SNE is set to 300. We have tried different perplexity (cf. Figs A-C in S1 File), but the results are quite similar. The seven classes can be further divided into four major classes (i.e., all alpha proteins, all beta proteins, alpha and beta proteins (a/b), and alpha and beta proteins (a+b)) and three minor classes (i.e., multi-domain proteins, membrane and cell surface proteins and peptides, and small proteins). All methods do not perform well on three minor classes. The potential reason is that the classification standard of SCOPe is not fully related to the structural similarity. Geometrically similar but differently classified proteins are not uncommon, and they are known as the cross fold similarities [9,16,46]. For example, a protein belonging to multi-domain proteins may contain a domain belonging to all alpha proteins and a domain belonging to all beta proteins. Descriptors of four major classes derived from GraSR, SGM and DeepFold are well separated as shown in the Fig 6, and the results can explain for their good performance. There is much overlap among different classes based on the descriptors of SSEF, which can explain for its unsatisfactory performance on both ranking and classification tasks.

An interesting observation is that descriptors of GraSR tend to form many small clusters instead of less but larger clusters like other methods. A potential reason is that structures of the same class can be further divided into different folds / superfamilies and structures of different folds / superfamilies may share low structural similarities.

### GraSR learns hidden information related to alignment implicitly

Due to our GNN-based encoders, the final descriptors of GraSR are derived by aggregating all node embeddings in the readout layer. Thus, we can extract these node embeddings as residue-level descriptors. The descriptor of each residue represents the local geometric features in its spatial neighborhood. We superimpose two protein structures based on their residue-level descriptors.

Given two protein structures, the cosine similarity matrix $C={\left[c_{ij}\right]}^{L_{1}\times L_{2}}\in {\left[-1,1\right]}^{L_{1}\times L_{2}}$ is calculated between each residue pairs, where *L*_1_ and *L*_2_ are the number of residues in the respective structures and *c*_*ij*_ denotes the cosine similarity between the *i*^th^ residue in one structure and the *j*^th^ residue in the other one. Then, Needlman-Wunsch dynamic programming algorithm is used to align two protein sequences [19]. The cosine similarity matrix serves as the scoring matrix. The penalty for opening and extending a gap is 0 and 0.1, respectively. Kabsch algorithm is used to find the optimal rotation and translation for two structures after determining the residue correspondence [47]. Other more complicated and effective alignment algorithms are not applied here since we only want to know whether GraSR could find an acceptable residue correspondence instead of obtaining the best superposition.

**Fig 7:**
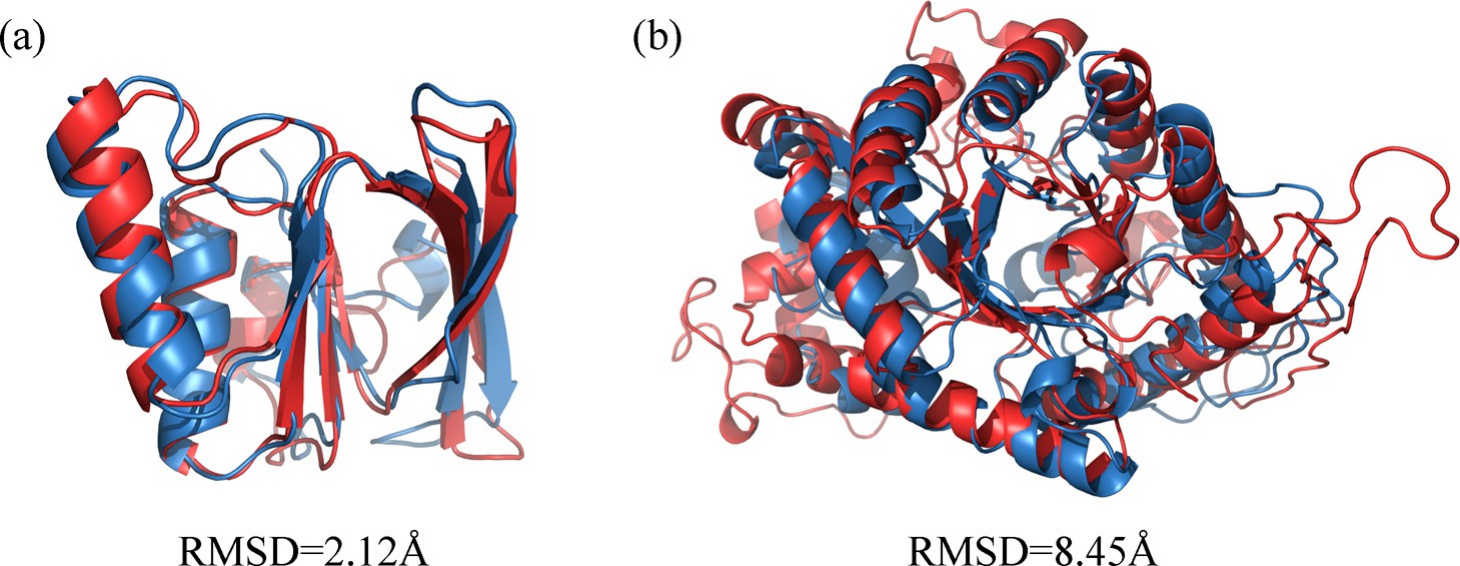
Protein structure superposition derived from the residue-level descriptors of GraSR. (A) SCOPe-sid: d1v59a2 (red) and d1h6va2 (blue) (B) SCOPe-sid: d5dqpa_ (red) and d1ezwa_ (blue).

Two proteins in Fig 7A belong to the same SCOPe family: FAD/NAD-linked reductases, N-terminal and central domains (SCOPe-sccs: c.3.1.5). The RMSD of their superposition is only 2.12Å. Proteins in Fig 7B belong to the same superfamily: Bacterial luciferase-like (SCOPe-sccs: c.1.16), but are in different families. Thus, the RMSD of them is relatively larger. The alignment has also been done to many other structures and their structural neighbors in the SCOPe and similar phenomena are also observed. Some exceptions do exist when the number of residues in two aligned proteins are varied. However, the overall results show that equivalent residues in two similar protein structures can be found based on the residue-level descriptors derived from GraSR. It should be noticed that GraSR only knows whether a protein structure pair is more similar than other ones and no alignment information is used in the training stage. Thus, the GNNs used in GraSR learn accurate node embeddings, which help determine the residue correspondence and benefit the global descriptors of the protein structures.

### Future directions

Although GraSR and other alignment-free methods can provide fast and accurate structure retrieval, there still exists space for further improvement. Structure alignment is not needed by alignment-free methods, which accelerates the comparison process; on the other hand, it is difficult for alignment-free methods to obtain the superposition information of the protein structures, which will be useful for understanding the structural similarity at the atomic level. The accuracy of alignment-free methods is inferior to alignment-based methods in some cases. The potential reason is also the lack of structure alignment. Combining these two approaches would be promising than using them alone. Faster alignment-free methods can be used to retrieve a small subset of roughly similar structures and then accurate but slower alignment-based methods are used to pick the most similar structures from the subset at atomic level. In addition, with further development of deep learning theory, it is possible and also necessary to extract more accurate superposition information from neural networks according to our observation in the section 4.2.

## Conclusions

Comparing the similarity of two protein 3D structure accurately in a fast manner is highly desired with the explosion of protein structure data. This will help us know more about the protein fold type space. In this study, we propose an effective protein structure representation learning method GraSR for this task, which is constructed under an effective contrastive learning framework. The encoders are redesigned and two novel strategies are proposed to further improve the performance. The encoders integrate a biLSTM and multiple graph convolutional layers to extract high-level features from both the primary structure and tertiary structure. The length-scaling cosine distance is designed to bridge the gap between the standard cosine distance and TM-score, and the dynamic training data partition strategy helps the encoder to learn more fine-grained relationship between protein structures. In addition, we propose a more comprehensive evaluation measurement named Top-K hit ratio, which can be seen as an extension to the Top-K accuracy. All protein structure representation methods are evaluated on the SCOPe v2.07 and the independent test set built from PDB. Compared with the existing methods, the result demonstrates that GraSR learns more discriminative protein structural descriptors and achieves higher performance on the ranking task and multi-class classification task. In addition to the global structure representation, GraSR can implicitly learn residue-level representations, which can be used to describe the local geometric features. We expect that the structural descriptors learned by GraSR could be useful for downstream tasks, such as structure-based protein function prediction and protein-ligand binding affinity prediction.

## Supporting information

S1 File Supplemental control experiments, analyses and figures. **Text A:** Rules for data filtering. **Text B:** The Architecture of the CNN-based encoder. **Text C:** Selection of K in dynamic training data partition. **Text D:** The distance-based feature is invariant to rotation and translation. **Text E:** Visualization by t-SNE at different perplexity. **Table A:** Performance of GraphFold when selecting different K in the dynamic training data partition on SCOPe v2.07. **Fig A:** Visualization of descriptors learned from GraSR and other methods by t-SNE (perplexity = 30). **Fig B:** Visualization of descriptors learned from GraSR and other methods by t-SNE (perplexity = 100). **Fig C:** Visualization of descriptors learned from GraSR and other methods by t-SNE (perplexity = 500).
(PDF)
