# Supplementary material for: Fast protein structure comparison through effective representation learning with contrastive graph neural networks
Source: PLoS Comput Biol. 2022 Mar 24;18(3):e1009986. doi: 10.1371/journal.pcbi.1009986 (PMC8982879; doi:10.1371/journal.pcbi.1009986)
Supplement: S1 File — Text A: Rules for data filtering. Text B: The Architecture of the CNN-based encoder. Text C: Selection of K in dynamic training data partition. Text D: The distance-based feature is invariant to rotation and translation. Text E: Visualization by t-SNE at different perplexity. Table A: Performance of GraphFold when selecting different K in the dynamic training data partition on SCOPe v2.07. Fig A: Visualization of descriptors learned from GraSR and other methods by t-SNE (perplexity = 30). Fig B: Visualization of descriptors learned from GraSR and other methods by t-SNE (perplexity = 100). Fig C: Visualization of descriptors learned from GraSR and other methods by t-SNE (perplexity = 500). (PDF) [file pcbi.1009986.s001.pdf]

Supporting Information

Fast Protein Structure Comparison through Effective Representation Learning with Contrastive Graph Neural Networks

CONTENTS

A. Rules for data filtering 2
B. The Architecture of the CNN-based encoder 2
C. Selection of K in dynamic training data partition 2
D. The distance-based feature is invariant to rotation and translation 2
E. Visualization by t-SNE at different perplexity 4
Reference 6

A. Rules for data filtering

SCOPe v2.07 is used to train and validate our model. 13 domains are removed for one of the following two reasons:

1. The PDB file only contains the coordinates of C_α atoms.

2. The PDB file contains multiple chains and at least one atom in it has alternate location. The reason is the TM-align program may miss some atoms when comes to this kind of PDB files.

Totally 14,310 domains are used for training our model. There are 1,045 additional domains are removed because other programs for comparison cannot generate descriptors from them. Therefore, totally 13,265 domains are used for the validation stage of cross-validation.

B. The Architecture of the CNN-based encoder

The architecture of CNN-based encoder is similar to the one used in DeepFold [1]. The input is the intra-residue distance matrix of a protein. The neural network contains 6 convolutional layers. The number of convolutional kernels are [128, 256, 512, 512, 512, 400] and the stride is 2. Relu and dropout layers (dropout rate is 0.5) are used after each convolutional layer. Comapred with DeepFold, two changes are made to avoid numerical error. The first one is that the kernel size is changed to [11, 3, 3, 3, 3, 3]; the second one is that batch normalization is substituted by instance normalization after each convolutional layer [2]. Then, the diagonal of each feature map is extracted and the mean of it is computed. Finally, a 400-d vector is output as the descriptor.

C. Selection of *K* in dynamic training data partition

In the dynamic training data partition, Top *K* percent structures are used to construct the subset $\mathbb{S}$. 5-flod cross-validation on SCOPe v2.07 is used to determine the *K*. The results in the Table A show that our model is not sensitive to the selection of *K*. There is no significant difference between *K*=10% and *K*=30%. The performance is slightly improved when we change *K*=50% to *K*=30%. Thus, *K* is set to 30%.

Table A. Performance of GraphFold when selecting different K in the dynamic training data partition on SCOPe v2.07

K percent	Avg. AUROC	Avg. AUPRC	Top-1	Top-5	Top-10
10%	0.9819	0.6568*	0.7268	0.7091	0.7395
30%	0.9823	0.6595	0.7282	0.7101	0.7400
50%	0.9809*	0.6506**	0.7215*	0.7942*	0.7366*

**p*-value of t-test is < 0.05; ***p*-value of t-test is < 10^{-4}; ****p*-value of t-test is < 10^{-9}.

D. The distance-based feature is invariant to rotation and translation

In this study, each residue in the protein is represented as a node in a graph. The distance-based

feature vector of any node in the node set $V = \{\mathbf{v}_1, \mathbf{v}_2, \dots, \mathbf{v}_k, \dots, \mathbf{v}_{N_r}\}$ contains T elements, where $\mathbf{v}_k$ is the Cartesian coordinate of kth residue in the protein sequence and N_r is the number of residues in the protein. Let's extract the tth element from it without loss of generality and denote it as $x^{(t)} = \left\|\mathbf{v}_k - \mathbf{p}_{ref}^{(t)}\right\| \, (t \le T)$. $\mathbf{p}_{ref}^{(t)}$ is the tth reference point and is defined as

$$\mathbf{p}_{ref}^{(t)} = \frac{1}{j-i} \sum_{\mathbf{v} \in V_{i:j}} \mathbf{v},$$

where $V_{i:j} = \{\mathbf{v}_i, \mathbf{v}_{i+1}, \dots, \mathbf{v}_{j-2}, \mathbf{v}_{j-1}\} \, (i < j)$.

Then, we denote the node set after rotation and translation as $V' = \{\mathbf{v}_1', \mathbf{v}_2', \dots, \mathbf{v}_k', \dots, \mathbf{v}_{N_r}'\}$. If we denote the rotation matrix as $\mathbf{R}$ and translation vector as $\mathbf{e}$, $\mathbf{v}_k' = \mathbf{R} \cdot \mathbf{v}_k + \mathbf{e}$. Similarly, the distance-based feature after rotation and translation can be denoted as $x^{(t)'} = \left\|\mathbf{v}_k' - \mathbf{p}_{ref}^{(t)\,'}\right\|$. We will prove $x^{(t)} = x^{(t)'}$.

Proof:

$$\begin{aligned} x^{(t)'} = \left\|\mathbf{v}_k' - \mathbf{p}_{ref}^{(t)\,'}\right\| &= \left\|\mathbf{v}_k' - \frac{1}{j-i} \sum_{\mathbf{v}' \in V_{i:j}'} \mathbf{v}'\right\| \\ &= \left\|\mathbf{R} \cdot \mathbf{v}_k + \mathbf{e} - \frac{1}{j-i} \sum_{\mathbf{v} \in V_{i:j}} (\mathbf{R} \cdot \mathbf{v} + \mathbf{e})\right\| \\ &= \left\|\mathbf{R} \cdot (\mathbf{v}_k - \frac{1}{j-i} \sum_{\mathbf{v} \in V_{i:j}} \mathbf{v})\right\| \\ &= \left\|\mathbf{R} \cdot (\mathbf{v}_k - \mathbf{p}_{ref}^{(t)})\right\| \\ &= \sqrt{(\mathbf{v}_k - \mathbf{p}_{ref}^{(t)})^{\mathrm{T}} \mathbf{R}^{\mathrm{T}} \mathbf{R} \cdot (\mathbf{v}_k - \mathbf{p}_{ref}^{(t)})} \end{aligned}$$

Let's recall that any rotation matrix is an orthogonal matrix. Thus $\mathbf{R}^{\mathrm{T}}\mathbf{R} = \mathbf{I}$ and

$$x^{(t)'} = \sqrt{(\mathbf{v}_k - \mathbf{p}_{ref}^{(t)})^{\mathrm{T}} (\mathbf{v}_k - \mathbf{p}_{ref}^{(t)})} = \left\|\mathbf{v}_k - \mathbf{p}_{ref}^{(t)}\right\| = x^{(t)}.$$

■

E. Visualization by t-SNE at different perplexity

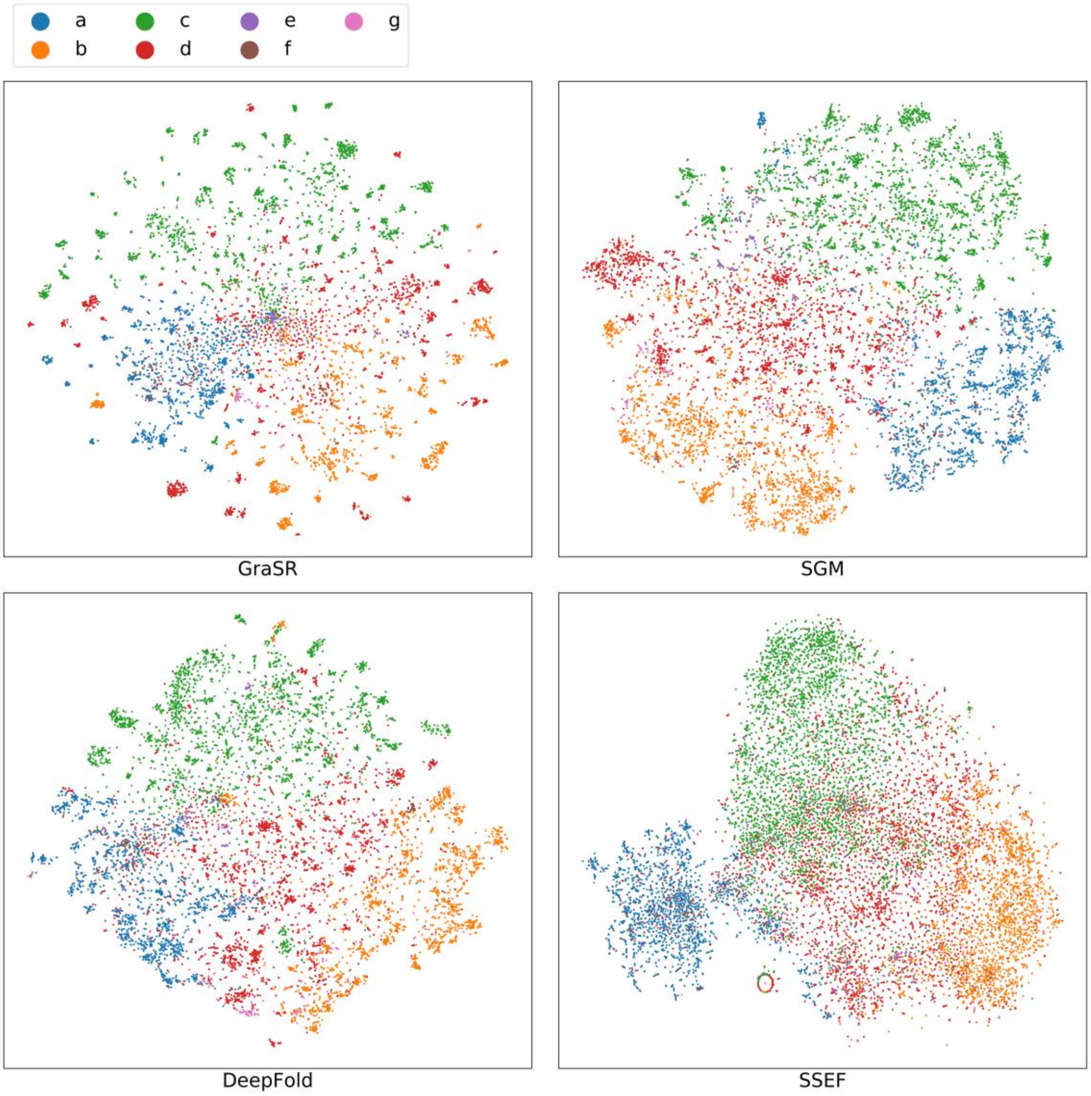

Fig A. Visualization of descriptors learned from GraSR and other methods by t-SNE (perplexity=30). a: All alpha proteins; b: All beta proteins; c: Alpha and beta proteins (a/b); d: Alpha and beta proteins (a+b); e: Multi-domain proteins (alpha and beta); f: Membrane and cell surface proteins and peptides; g: Small proteins.

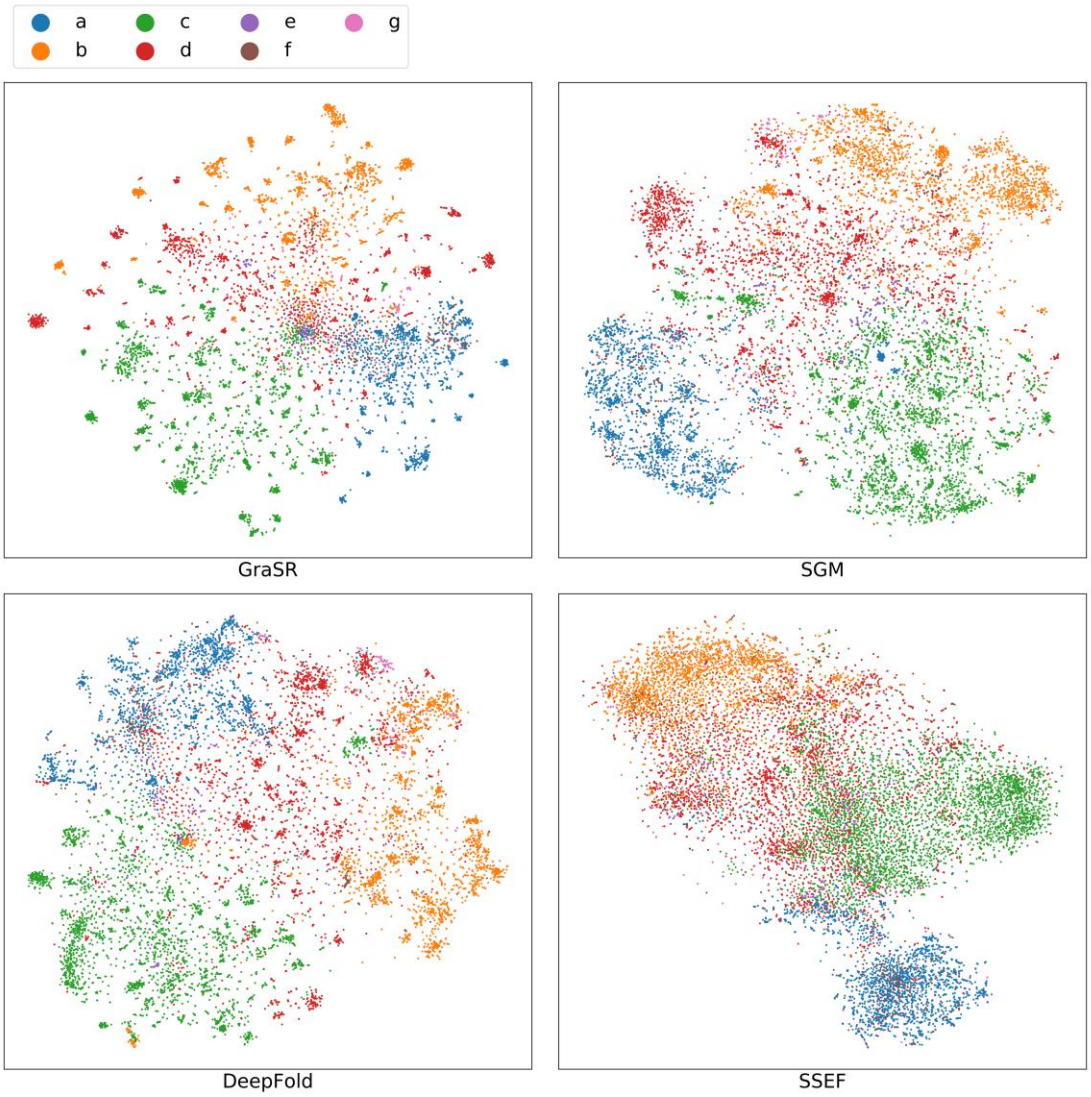

Fig B. Visualization of descriptors learned from GraSR and other methods by t-SNE (perplexity=100). a: All alpha proteins; b: All beta proteins; c: Alpha and beta proteins (a/b); d: Alpha and beta proteins (a+b); e: Multi-domain proteins (alpha and beta); f: Membrane and cell surface proteins and peptides; g: Small proteins.

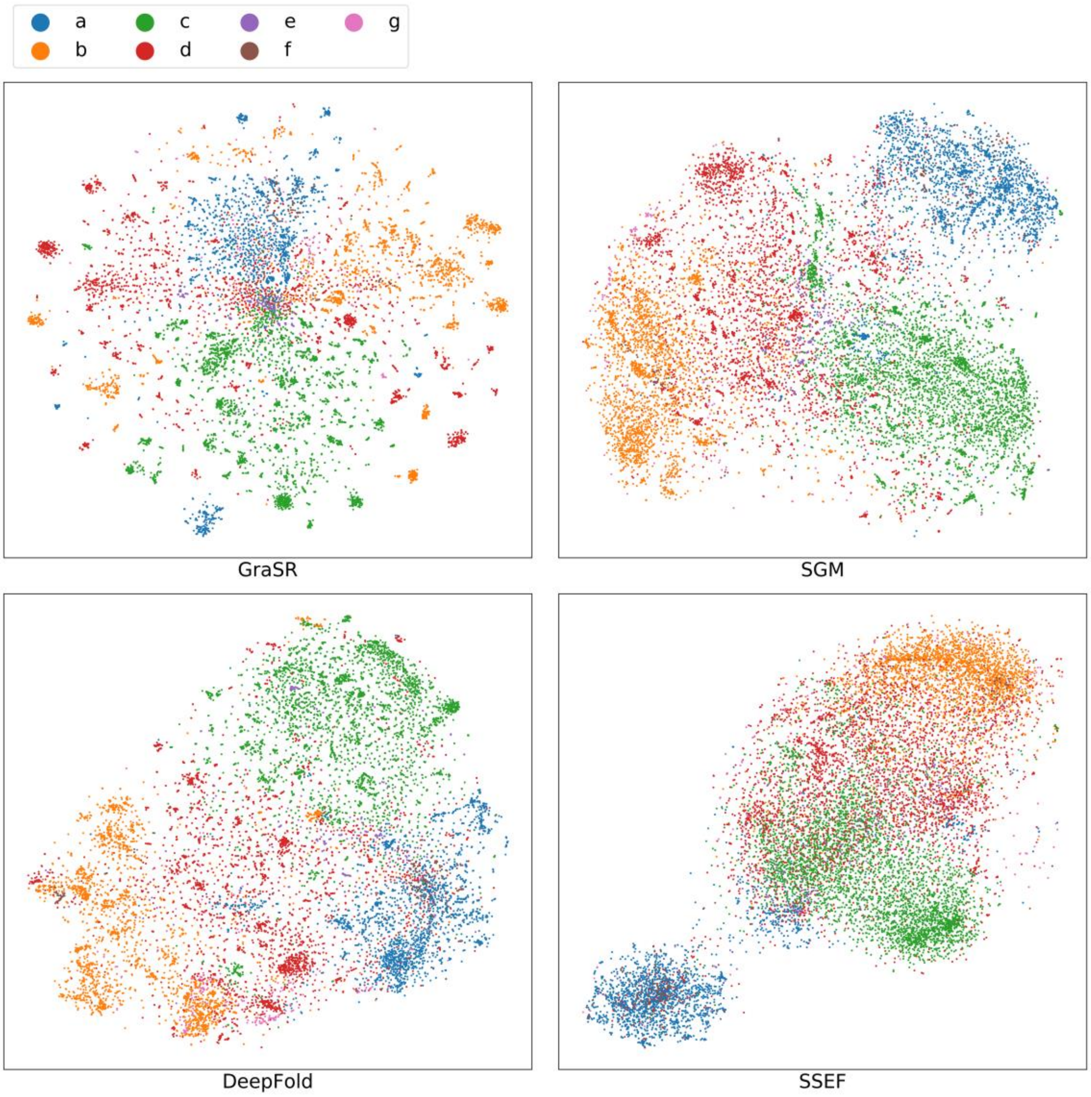

Fig C. Visualization of descriptors learned from GraSR and other methods by t-SNE (perplexity=500). a: All alpha proteins; b: All beta proteins; c: Alpha and beta proteins (a/b); d: Alpha and beta proteins (a+b); e: Multi-domain proteins (alpha and beta); f: Membrane and cell surface proteins and peptides; g: Small proteins.

Reference

[1] Liu, Y., et al., Learning structural motif representations for efficient protein structure search. Bioinformatics, 2018. 34(17): p. 773-780

[2] Ulyanov, D., et al., Instance normalization: The missing ingredient for fast stylization. arXiv, 2016. abs/1607.08022.
